# Upregulation of prolylcarboxypeptidase (PRCP) in lipopolysaccharide (LPS) treated endothelium promotes inflammation

**DOI:** 10.1186/1476-9255-6-3

**Published:** 2009-01-27

**Authors:** My-Linh Ngo, Fakhri Mahdi, Dhaval Kolte, Zia Shariat-Madar

**Affiliations:** 1 School of Pharmacy, Department of Pharmacology, University of Mississippi, Oxford, MS, USA; 2 School of Pharmacy, Department of Pharmacognosy, University of Mississippi, Oxford, MS, USA

## Abstract

**Background:**

Prolylcarboxypeptidase (*Prcp*) gene, along with altered PRCP and kallikrein levels, have been implicated in inflammation pathogenesis. PRCP regulates angiotensin 1–7 (Ang 1–7) – and bradykinin (BK) – stimulated nitric oxide production in endothelial cells. The mechanism through which kallikrein expression is altered during infection is not fully understood. Investigations were performed to determine the association between PRCP and kallikrein levels as a function of the upregulation of PRCP expression and the link between PRCP and inflammation risk in lipopolysaccharide (LPS)-induced endothelium activation.

**Methods:**

The *Prcp *transcript expression in LPS-induced human umbilical vein endothelial cells (HUVEC) activation was determined by RT-PCR for mRNA. PRCP-dependent kallikrein pathway was determined either by Enzyme Linked ImmunoSorbent Assay (ELISA) or by biochemical assay.

**Results:**

We report that PRCP is critical to the maintenance of the endothelial cells, and its upregulation contributes to the risk of developing inflammation. Significant elevation in kallikrein was seen on LPS-treated HUVECs. The conversion of PK to kallikrein was blocked by the inhibitor of PRCP, suggesting that PRCP might be a risk factor for inflammation.

**Conclusion:**

The increased PRCP lead to a sustained production of bradykinin in endothelium following LPS treatment. This amplification may be an additional mechanism whereby PRCP promotes a sustained inflammatory response. A better appreciation of the role of PRCP in endothelium may contribute to a better understanding of inflammatory vascular disorders and to the development of a novel treatment.

## Background

Prolylcarboxypeptidase (PRCP) dysfunction is associated with adverse cardiovascular consequences such as inflammation and hypertension [1,2]. Although the physiological role(s) of PRCP is still poorly understood, PRCP has been shown to be an active participant in processes such as cell permeability via the activation of prekallikrein (PK) and the melanogenic signaling pathway [3]. PRCP-dependent plasma prekallikrein activation influences the permeability of the endothelium by liberating bradykinin (BK) from a protein precursor, high molecular weight kininogen (HK). BK- mediated bradykinin B 2 receptor activation leads to the release of nitric oxide and prostaglandins [4,5]. In addition, PRCP metabolizes angiotensin II (Ang II) to angiotensin 1–7 (Ang 1–7) and angiotensin III (Ang III) to angiotensin 2–7 (Ang 2–7). Ang 1–7 -mediated Ang 1–7 receptor Mas activation causes the release of prostaglandins and nitric oxide[6]. Thus, PRCP regulates Ang 1–7 – and BK – stimulated nitric oxide production in endothelial cells, highlighting PRCP's role as a regulatory protease rather than a digestive protease.

Kallikrein (activated prekallikrein) is implicated in many physiological and pathological processes including the blood coagulation, the initiation of the classical complement cascade pathway, as well as activating the alternative complement pathway [7,8]. Kallikrein is also involved in induction of elastase release from neutrophils and conversion of prourokinase to urokinase to initiate fibrinolysis [9-12]. Kallikrein over-expression parallels endothelial lesion, tissue injury, and sepsis – underscoring the correlation between kallikrein alterations and inflammation [13-15]. The mechanism by which kallikrein expression is altered during infection is not fully understood; however, some possible mechanisms have been postulated by others [16-19].

Of interest, PK is markedly depressed immediately following intramural injection of exogenous bacterial components to Lewis rats or to normal human volunteers, an indicator of PK activation[20,21]. The reduction in PK levels has been attributed to the activated factor XII(FXIIa) -induced plasma kallikrein-kinin system (KKS) activation via factor XII autoactivation[20,21]. The autoactivation of factor XII is necessary step to make factor XII susceptible for cleavage by kallikrein to support activation of the KKS in plasma as described[22]. Interestingly, activation of PK is not abolished in patients with factor XII deficiency, suggesting that PK is activated by an uncharacterized mechanism[23]. Since PRCP (a PK activator) is also elevated during inflammation, we decided to develop an endothelium model of inflammation which would enable us to determine whether the upregulation of PRCP expression would cause an increase in the generation of kallikrein.

We document that the upregulation of PRCP in lipopolysaccharide (LPS) pretreated endothelial cells results in an increase kallikrein generation. The implication of this observation is that PRCP might be an independent risk factor for inflammation. Furthermore, the upregulation of PRCP expression might promote inflammation from an acute to a chronic state through Ang 1–7 – and BK – stimulated nitric oxide production. Inactivation of PRCP-dependent pathway becomes extremely important in clinical situations such as septic shock and systemic inflammation.

## Methods

### Materials

Agarose, ladder, 0.5 M EDTA, pH 8.0, ultra pure distilled water DNase, RNase free and dNTP were purchased from Gibco BRI (Invitrogen Life Technology, Carlsbad, CA). Prestained low molecular weight standards, nitrocellulose, and polyacrylamide were all purchased from Bio-Rad Corp (Richmond, CA). The bradykinin B2 receptor antagonist (HOE140, icatibant) was purchased from Peninsula Laboratories (San Carlos, CA). Markit BK kit was purchased from Dainippon Pharmaceutical (Osaka, Japan). H-D-Pro-Phe-Arg-p-nitroanilide (S2302) was purchased from Dia-Pharma (Franklin, OH).

### Enzymes, proteins, and biochemicals

Ribonucleotides, deoxyribonucleotides, and restriction enzymes were purchased from Roche Applied Science (Indianapolis, IN). RNasin Plus Ribonuclease inhibitor, RNase-free DNase I, and RNAgents total RNA isolation system were obtained from Promega (Madison, WI). Oligonucleotide primers for PCR were synthesized at Gibco BRI (Carlsbad, CA). Platinum-polymerase and taq-polymerase were purchased from Roche Applied Science.

Single chain HK (MW = 120 kDa on reduced SDS-PAGE) with a specific activity of 13 U/mg in acetate buffer (4 mM sodium acetate-HCl and 0.15 M NaCl, pH 5.3) was purchased from DiaPharma Laboratories, Inc. (West Chester, Ohio). Prekallikrein (PK) with a specific activity of 27 U/mg was purchased from Enzyme Research Laboratories (South Bend, IN).

### Culture of endothelial cells

Human umbilical vein endothelial cells (HUVEC) were purchased from Clonetics (San Diego, CA) and cultured according to the supplier's instructions. Cells displayed typical staining for the endothelial cell marker, von Willebrand factor (data not shown). Cellular morphology was typical for endothelial cells – monolayered 'cobblestone' morphology and the absence of contaminating fibroblasts. Cells were cultured on a 2 μg/well fibronectin substrate and passaged using 0.1% trypsin-0.02% ethylenediaminetetra-acetic acid (EDTA) followed by neutralizing trypsin. They were cultured in EGM medium purchased from Invitrogen Corp (Carlsbad, California) supplemented with 25 U/ml penicillin and 25 μg/ml streptomycin and 2% heat inactivated fetal calf serum purchased from Hyclone (Logan, UT). Cells at passage numbers one to five were used at confluence (>3.0 × 10^4^/cm^2^) 24 h after seeding. Lipopolysaccharide (E. coli serotype 0111:B4) was diluted in culture medium, and added to cells to final concentrations of 1–1000 ng/ml for 1–24 h.

### Lipopolysaccharide (LPS) – induced HUVEC activation

These experiments were performed to determine the sub-lethal dose of LPS that activates HUVECs. To determine LPS-induced cytotoxicity, HUVECs were incubated with various concentrations of LPS over time. At the end of stimulation, the lactate dehydrogenase (LDH) release was performed to test for the loss of plasma membrane integrity by using a LDH diagnostic kit as outlined in the manufacture's instructions. A hallmark of apoptosis is the onset of DNA fragmentation. Apoptotic cells were determined by terminal deoxynucleotidyl transferase-mediated dUTP nick end-labeling (TUNEL) assay under fluorescence microscopy. Cells on the slides were fixed in a solution containing 4% paraformaldehyde and 3% H_2_O_2 _for 10 min followed by washing with buffer containing 0.5% Triton X-100 at 4°C for 2 min. The slides were then incubated with TUNEL reactions at 37°C for 60 min and then in peroxidase (POD) at 37°C for 30 min. Finally, cells were stained with DAB (3,3'-diaminobenzidine-tetrahydrochloride) and studied microscopically.

### Prekallikrein activation on LPS-stimulated endothelial cells

LPS (0.3 μg/ml) pretreated confluent monolayers of HUVEC at 4 × 10^4 ^cells density/well in microtiter plate cuvette wells were washed three times with HEPES-NaHCO_3 _buffer [137 mM NaCl, 3 mM KCl, 12 mM NaHCO_3_, 14.7 mM Hepes, 5.5 mM Dextrose and 0.1% gelatin, pH 7.4] containing 2 mM CaCl_2_, and 1 mM MgCl_2 _and blocked with 1% gelatin for 1 h at 37°C. After blocking, 20 nM HK in the same buffer was added to the monolayers for 60 min at 37°C. At the end of incubation, the cells were washed and then incubated with PK (20 nM) in HEPES- NaHCO_3 _buffer for 60 min at 37°C. Cells were washed and 0.8 mM H-D-Pro-Phe-Arg-p-nitroanilide (S2302) (Dia-Pharma, Franklin, OH) was added in the same buffer and substrate hydrolysis proceeded for 1 h at 37°C. The rate of paranitroanilide liberation from H-D-Pro-Phe-Arg-paranitroanilide (0.8 mM, S2302) by kallikrein was determined by the absorbance at 405 nm [24]. Additional experiments were performed to determine if increasing concentration of Z-pro-prolinal (PRCP inhibitor) inhibited PK activation.

### Measuring the generation of bradykinin by PRCP-dependent PK activation pathway on LPS-treated HUVECs

The generation of BK by PRCP-dependent pathway was monitored in order to assess the physiological and pathophysiological role of PRCP. In these experiments, HUVECs, LPS-treated HUVECs, or PRCP-siRNA transfected HUVECs pretreated with LPS were treated with 1 μM HOE140, and 100 μM lisinopril to block the metabolism of BK by bradykinin B2 receptors and angiotensin converting enzyme. After 5 minutes of incubation, cells were incubated with HEPES-NaHCO_3 _buffer containing 50 nM HK, 50 nM PK in the absence or presence of 1 μM HKH20 or Z-pro-prolinal. After 1 h of incubation, the supernatants of these reactions were collected and either immediately deproteinized with trichlororacetic acid or frozen at -80°C for further study. BK in the samples was determined using a commercial kit (Markit BK, Dainippon Pharmaceutical; Osaka, Japan), performed according to the supplier's instructions.

### Permeability determination for the plasma KKS

*In vitro *cell permeability assay was performed according to the manufacturer's protocol (CHEMICON, Billerica, MA). Briefly, endothelial cells (1.0 × 10^6 ^cells/ml) were subcultured in the inserts of permeability chambers that were coated with collagen. Cells were incubated in the tissue culture incubator at 37°C until they well reached 100% confluency. Then, the endothelial cell monolayer were incubated with 300 nM HK, 300 nM PK, or the complex of HK and PK (300 nM each), 300 nM bradykinin, or 0.3 μg/ml LPS for 3 hours at 37°C in the tissue culture incubator. At the end of incubation, 150 μl of FITC-Dextran (1:30 dilution) was added to each insert for 5 min at room temperature, and then 100 μl of the solution in the lower chamber was transferred to a 96-well plate. The plate was read in a Perkinelmer (precisely) Envision 2103 Multimode Reader at excitation wavelength of 480 nm and emission wavelength of 530 nm and with the bandwidth of 10. Control inserts with cells plated were treated with HEPES carbonated buffer and used as a control.

### Small interfering RNA

The 19-nt siRNA duplex (5'-GACUCCUCUGGUUGAUCAUTT-3') used were designed to recognize human PRCP transcript, and was synthesized at Integrated DNA Technologies. These unique nucleotide residues within the PRCP had no identity with known mammalian genes. Transfection of siRNA into HUVEC was carried out in a six-well plate using lipofectamine 2000 according to the manufacturer's instructions (Invitrogen, Carsbad, CA) and as described [25]. Two microliters of lipofectamine 2000 were diluted in 50 μl of Opti-MEM, and the mixture was incubated for 5 min at room temperature. For each well, 2 μl of siRNA (20 μM) were diluted in 50 μl of Opti-MEM mixed and incubated for 25 min at room temperature. The siRNA was added to the lipofectamine 2000 solution and mixed. The transfection mixture was added to each well containing 5 × 10^4 ^endothelial cells in suspension and transfections were incubated for 48 h in the incubator at 37°C, 5% CO_2_. Then, PK activation was determined on PRCP-siRNA transfected HUVEC.

### Gene expression studies

To define the inflammatory properties of HUVECs, a panel of nine biomarkers of activated endothelium participating in thrombophilia, fibrinolysis, endothelial viability, and inflammation was analyzed in LPS-treated HUVECs. Total RNA from untreated as well as LPS-treated HUVEC were isolated and the expression levels of the endothelial risk factors were analyzed by RT-PCR.

We further determined PRCP expression in LPS-induced endothelium activation. The objective of this investigation was to determine whether LPS mediates a concentration- and time-dependent increase in the rate of PRCP synthesis in LPS-treated endothelial cells. For analyses of PRCP, endothelial nitric oxide synthase (eNOS), von Willebrand factor (VWF), tissue plasminogen activator (tPA), plasminogen activator inhibitor 1 (PAI-1), bradykinin B1 receptor (BKB1R), bradykinin B2 receptor (BKB2R), intercellular adhesion molecule-1 (ICAM-1), and glyceraldehydes-3-phosphate dehydrogenase (GAPDH) genes expression levels, RNA was extracted from cell monolayers using Trizol (Invitrogen Corp., Carlsbad, CA) in accordance with the manufacturer's specifications. RNA was then treated with DNAse I (Ambion Inc., Austin, TX) to eliminate genomic DNA contamination. cDNA was derived from HUVEC exposed to LPS to give expression levels of the genes of interest using Super Script II or III RNase H-Reverse Transcriptase (Invitrogen Corp.).

Primers used in the RT-PCR analyses were designed based on published gene sequences. Annealing temperatures used for all were 60°C. PCR product length in base pairs (bp) is indicated, and all PCR products were isolated, sequenced, and assessed against published human sequences using NCBI Blast to confirm they represented products from the genes of interest. The list of primers used in our investigations is tabulated in Table 1.

**Table 1:** Primer pairs used to analyze the expression of nine biomarkers of coagulation activation, fibrinolysis, endothelial injury, and inflammation.

	**Sense primer**	**Position On cDNA**	**Antisense primer**	**Position on cDNA**	**PCR Product Length** **(bp)**
**PRCP**	5'-ATGGGCCGCCGAGCCCTCCTG-3'	114–134	5'-GGTTGGTTGGCAAGTGTAGG-3'	225-206	111
**BKB1R**	5'-CACTTTGCAAGGATGGTGGAGTTG-3'	764–787	5'-GGAGGCCAGGATGTGATAGTTGAA-3'	853-830	89
**BKB2R**	5'-CTGGGTGTTTGGAGAGGTGT-3'	479–499	5'-ACGAGCATCAGGAAGCAGAT-3'	565-545	86
**tPA**	5'-GGCTGTGGACAGAAGGATGT-3'	1676–1695	5'-TGCACTCTTCCCTCTCCTGT-3'	1909-1890	233
**PAI-1**	5'-CAGACCAAGAGCCTCTCCAC-3'	1073–1092	5'-GACTGTTCCTGTGGGGTTGT-3'	1254-1235	181
**ICAM-1**	5'-GGCTGGAGCTGTTTGAGAAC-3'	644–663	5'-ACTGTGGGGTTCAACCTCTG-3'	845-826	201
**vWF**	5'-TGCTGACACCAGAAAAGTGC-3'	3343–3362	5'-AGTCCCCAATGGACTCACAG-3'	3539-3520	196
**eNOS**	5'-AGCATCCCTACTCCCACCA-3'	419–437	5-'ACCTCCCAGTTCTTCACACG-3'	520-501	101
**GAPDH**	5'-GAGTCAACGGATTTGGTCGT-3'	122–141	5'-GACAAGCTTCCCGTTCTCAG-3'	306-287	184

Primers were designed based on the cDNA sequences of human genes.

### Statistical analyses

Results are expressed as mean ± SEM, and data was analyzed using Student's t-test for significant difference. Statistical significance was defined as P < 0.05.

## Results

### Characterizing endothelium model of inflammation

PRCP expression is up-regulated during inflammation [2,3]. Emerging evidence suggests that lipopolysaccharide (LPS) activates the plasma kallikrein-kinin system in the choroid plexus [26]. The mechanism by which kallikrein expression is altered during infection is not fully understood. In endothelium, PRCP converts prekallikrein (PK) to kallikrein [24]. We decided to develop an endothelium model of inflammation, which would enable us to assess whether the upregulation of PRCP expression would cause an increase in kallikrein generation.

To develop cell model of inflammation, human umbilical vein endothelial cells (HUVEC) were treated with LPS. A hallmark of apoptosis is the onset of DNA fragmentation. To determine the sub-lethal dose of LPS, we used terminal deoxynucleotidyl transferase-mediated dUTP nick end labeling (TUNEL) technique to assess DNA fragmentation during apoptosis. 44% of cells incubated with 25 μg/ml LPS for 3 h showed brown grains (typical features of apoptosis) after incubating with DAB (3,3' -diaminobenzidine-tetrahydrochloride) (Table 2).

**Table 2:** LPS-induced apoptosis of endothelial cells.

**Lipopolysaccharide** **(μg/ml)**	**Ratio of apoptosis** **(%)**
Control	4.0 ± 1.0
1	5.0 ± 2.0
10	8.0 ± 2.0
25	44 ± 3.0

To determine cell viability, the lactate dehydrogenase (LDH) release (Promega, Madison, WI) was measured. LDH release assay showed that the endothelium plasma membrane integrity was intact after the LPS (0.2 μg/ml) stimulation for 16 h. Cell viability assay along with phenotypic observation of apoptosis under microscopy suggested that 1 μg/ml LPS stimulated cells did not induce cell apoptosis.

von Willebrand factor (VWF) expression is up-regulated during endothelial cell activation. Since the concentration of LPS in plasma or blood of patients with sepsis is about 200 ng/ml, we treated HUVEC with LPS (0.2 μg/ml) at different times (1, 2, 3, 8, 16, and 24 h) to determine VWF upregulation. VWF transcript was significantly increased in LPS – treated HUVEC within 16 h suggesting the activation of HUVEC. Thus, the time point (16 h) and dosage (0.2 μg/ml) became the standard in subsequent experiments.

To further define the inflammatory properties of HUVECs, a panel of nine biomarkers of activated endothelium participating in thrombophilia, fibrinolysis, endothelial viability, and inflammation was analyzed in LPS-treated HUVECs. Total RNA from untreated as well as LPS-treated HUVEC was isolated, and the expression levels of the endothelial risk factors were analyzed by RT-PCR (Table 3). Significant elevation of the mRNA of bradykinin B 1 receptor (BKB1R), ICAM 1, VWF, PAI-1 was present in LPS-treated endothelial cells within 16 h. However, the mRNA of bradykinin B 2 receptor (BKB2R) and eNOS was slightly decreased. These data suggested that 0.2 μg/ml LPS would transform quiescent HUVECs into an inflammatory stage.

**Table 3:** Total RNAs from untreated and LPS-treated endothelial cells were isolated and reverse transcribed.

**Biomarker** **(mRNA)**	**RT-PCR products Intensity (arbitrary Unit)**	
	
	**HUVEC**	**LPS-HUVEC**
BKB1R	0	57
BKB2R	320	234
AT1	127	258
AT2	340	560
tPA	243	340
PAI-1	402	905
VWF	297	844
ICAM-1	0	97
eNOS	176	105

PCR products were resolved in 1% agarose gel and detected by ethidium bromide. The intensity of PCR products were quantified after normalizing to GAPDH. Bradykinin B1 receptor; BKB1R, Bradykinin B2 receptor; BKB2R, angiotensin type 1 receptor; AT1, angiotensin type 2 receptor; AT2, tissue plasminogen activator; tPA, plasminogen activator inhibitor 1; PAI-1, von Willebrand factor; VWF, intercellular adhesion molecule-1; ICAM, endothelial nitric oxide synthase; eNOS.

### LPS enhances prekallikrein activation on HUVEC

Previous investigations have suggested that the kallikrein-kinin system [KKS, the heteromeric dimer of HK and PK] of blood coagulation contributes to thrombogenicity of atherosclerotic plaque as well as angiogenesis in inflammation and cancer [27-29]. We analyzed the response of PRCP-dependent PK pathway in endothelial cells exposed to LPS by using combined molecular and biochemical approaches.

**Figure 1:**
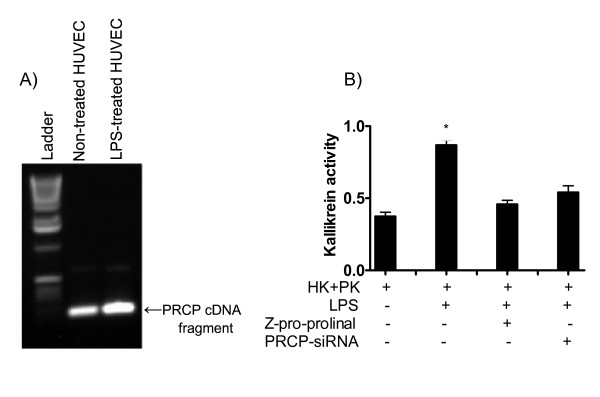
**LPS enhances prekallikrein activation and PRCP expression in HUVEC**. **Panel A: **total RNA was isolated from HUVEC and LPS treated HUVEC (LPS-HUVEC) cells and then amplified by RT-PCR. Amplified DNA (100 bp fragment) was resolved on a 1.5% agarose gel. **Panel B: **20 nM PK in the absence or presence of inhibitor was incubated with 20 nM HK bound to untreated, LPS-pretreated, or PRCP-siRNA transfected cells pretreated with LPS at 37°C. The liberation of paranitroanilide (pNA) from substrate (S2302) by kallikrein was measured at 405 nm. *p < 0.01 vs. untreated cells. The presented are the mean ± SEM of triplicate points of 10 independent experiments.

To test the effect of LPS on PRCP expression and activation, confluent monolayers of HUVECs were pretreated with LPS (0.2 μg/ml) for 16 h at 37°C. Using GAPDH as external control, the relative expression of PRCP mRNA in LPS-HUVEC was two-fold higher than in HUVEC (Figure 1A). The induction of *Prcp *tanscript in response to LPS is reported here for the first time. The mechanisms accounting for the robust PRCP expression seen in these cells remains speculative. PK activation on HK bound to HUVEC was significantly (p < 0.01) higher on LPS pretreated cells than on untreated cells. Z-Pro-Prolinal (0.7 mM) blocked PRCP-dependent PK activation by twofold (Figure 1B). Since a specific and an irreversible inhibitor of PRCP was not available, investigations were performed to determine the effect of PRCP-siRNA on PRCP-dependent PK activation. PK activation was reduced by 45% on PRCP-siRNA transfected cells. The modest reduction of PK activation on the PRCP-siRNA transfected cells could be due to poor transfection efficiency in HUVECs. However, our observations suggest that LPS potentiates PRCP expression and activity. The increase in PRCP activity led to a two-fold increase in the generation of kallikrein, which was blocked by z-Pro-Prolinal in LPS pretreated endothelial cells. These data raise the possibility that there is a causal relationship between PRCP expression and kallikrein generation.

### Bradykinin liberation on LPS-treated HUVEC

**Figure 2:**
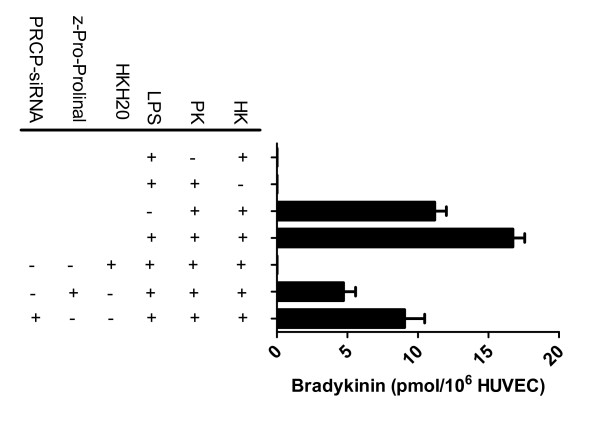
**PRCP-dependent prekallikrein (PK) activation and bradykinin (BK) liberation on LPS-treated HUVEC**. Untreated, LPS-pretreated, or PRCP-siRNA transfected cells pretreated with LPS were incubated with 100 nM HK alone or in the presence of 1 μM HKH20 in HEPES buffer containing 2 mM Ca^2+ ^and 1 mM Mg^2+ ^at 37°C for 60 min. Afterward, 100 nM PK with 1 μM lisinopril [angiotensin-converting enzyme inhibitor (ACE)] and 1 μM HOE 140 (bradykinin B2 receptor antagonist) was added and incubated with HUVEC at 37°C for 60 min in the same buffer. Forty-eight hours after transfection with 100 nM PRCP-siRNA or control, cells were incubated with HK and PK and assessed for BK generation as described above. After PK activation on HUVEC, the buffer from each of the wells was collected and deproteinized by treatment with trichloroacetic acid. The data are from three experiments (means ± SEM).

Investigations next proceeded to determine whether the upregulation of PRCP-dependent PK activation would lead to an increase in bradykinin (BK) generation on LPS-treated HUVEC. As shown in Figure 2, BK (11.2 × 10^6 ^pmol/10^6 ^HUVEC) was released into incubation buffer when the complex of HK/PK was assembled on HUVEC. However, LPS activated the PRCP-dependent PK pathway by enhancing liberation of BK (16.7 × 10^6 ^pmol/10^6 ^HUVEC) in HUVEC (Figure 2). The amount of BK generation was 40 percent higher on LPS-treated cells than on untreated cells. No BK was detected in the absence of added HK or PK. The extent of BK liberation from the assembly of PK on HK was abolished by the presence of HKH20 (HK cell binding site) suggesting that HK/PK binding to LPS-treated cells is essential in regulating endothelium function (Figure 2). Z-pro-prolinal (1 mM) inhibited the formation of BK by 60%.

By utilizing siRNA, we determined that PRCP plays a functional role in PK activation and bradykinin generation. We analyzed BK generation on cells transfected with PRCP-siRNA for 48 hours. As shown in Figure 2, downregulation of PRCP in cells transfected with 100 nM siRNA targeting PRCP resulted in 40–50% reduction in BK generation. The inability of siRNA to block BK levels by 100% might be due to the poor transfection efficiency in HUVEC. These results suggest that PRCP may contribute to the risk of developing inflammation.

### *In vitro *endothelial cell permeability

**Figure 3:**
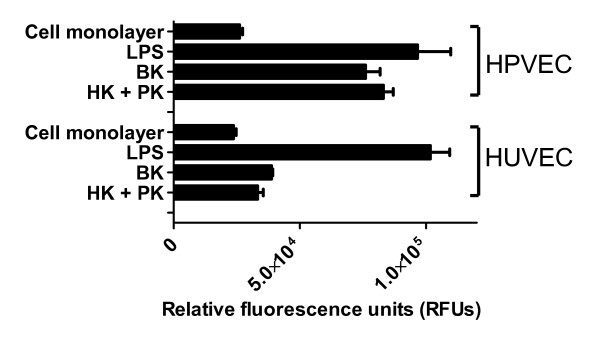
**Influence of the plasma kallikrein-kinin activation on endothelium monolayer permeability**. Endothelial cells (1.0 × 10^6 ^cells/ml) were seeded in the inserts of permeability chambers that were coated with collagen. The endothelial cell monolayer were incubated with 300 nM HK, 300 nM PK, or the complex of HK and PK (300 nM each), 300 nM bradykinin, or 0.3 μg/ml LPS for 3 hours at 37°C in the tissue culture incubator. Then, 150 μl of FITC-Dextran (1:30 dilution) was added to each insert and incubated for 5 min at room temperature. The presence of FITC-Dextran (1:30 dilution) in the lower chamber was determined by a Perkinelmer (precisely) Envision 2103 Multimode Reader at excitation wavelength of 485 nm and emission wavelength of 530 nm.

Having established that LPS potentiates PRCP expression and subsequently causes an increase in BK generation, we next determined if this process would influence endothelial cell permeability. The effects of bradykinin, HK, PK, or the HK-PK complex on both HUVEC permeability and on human pulmonary vein endothelial cell (HPVEC) permeability were determined by quantifying the permeability of FITC-Dextran through the cell monolayer. As shown in Figure 3, the complex of HK-PK (300 nM each, physiologic concentration), BK (300 nM), or LPS (2 μg/ml) increased endothelial monolayer permeability. No detectable permeability was seen when HUVEC or HPVEC monolayer was treated with buffer containing FITC-Dextran, indicating the occlusion of the membrane pores by the endothelial monolayer. Using a different permeability assay, others also have shown that addition of BK could cause a significant increase in permeability to fluorescein isothiocyanate-labeled human serum albumin in HUVEC[30,31]. As shown in Figure 3, the induction of cell permeability by BK or the HK-PK complex in HUVEC was lower than in HPVEC by two orders of magnitude, under our experimental conditions. The cellular basis for the differing cell permeability responses of HUVEC and HPVEC is not known. However, we cannot exclude the possibility that the expression of BK receptor subtypes on HUVEC is different than that of HPVEC, because such an observation has been described on other endothelial cell types[32]. In primary HUVECs and HPVEC, 2 μg/ml LPS significantly increased the permeability of FITC-Dextran through the cell monolayer. The robust permeability of FITC-Dextran through the cell monolayer by LPS might be due to cell detachment as suggested by Bannerman[33]. Neither HK – nor PK- induced permeability of FITC-Dextran through the monolayer of HUVEC or HPVEC (data not shown). These findings indicate that PRCP enhances cell monolayer permeability through activation of plasma kallikrein-kinin system which generates BK.

## Discussion

Prolylcarboxypeptidase (PRCP) activates prekallikrein (PK) to kallikrein leading to the generation of bradykinin (BK) from high molecular weight kininogen (HK)[24]. *Prcp *gene along with altered PRCP and kallikrein levels have been implicated in inflammation pathogenesis. PK is significantly depressed immediately following intramural injection of exogenous bacterial components to Lewis rats or to normal human volunteers suggesting the potential activation of PK to kallikrein by the activated factor XII [20,21]. However, the activation of PK is not abolished in patients with factor XII deficiency, suggesting that PK is activated by an uncharacterized mechanism[23]. The mechanism by which kallikrein expression is altered during infection is not fully understood.

The aim of the present study was to determine the association of PRCP and kallikrein levels as a function of the upregulation of PRCP expression and the link between PRCP and inflammation risk in lipopolysaccharide (LPS)-induced endothelium activation. The major finding of our current investigation was that the stimulation of endothelial cells by LPS resulted in a significant upregulation of PRCP mRNA expression. The activation of PK to kallikrein was also enhanced on LPS-treated HUVECs. The amount of BK generation was significantly higher on LPS-treated cells than on untreated cells. PRCP enhanced cell monolayer permeability through activation of plasma kallikrein-kinin system which generates BK. The down-regulation of PRCP by PRCP-siRNA markedly blocked both kallikrein and bradykinin (BK) generation on LPS-pretreated HUVECs. Thus, the present study extends the role of PRCP-dependent PK activation into inflammatory reactions.

Physiologically, BK is a cardioprotective peptide. However, uncontrolled BK – stimulated nitric oxide production could promote endothelial dysfunction. Experiments were performed to determine if PRCP-dependent PK activation would result in an increase in BK generation on LPS-treated HUVECs. BK generation was significantly higher on LPS-pretreated cells than on untreated cells indicating that PRCP modulates BK generation. The present findings suggest that the upregulation of PRCP can lead to an increase in BK generation in response to LPS-induced endothelial cell activation, and we therefore believe that PRCP might promote inflammatory response. PRCP inhibitors could represent a novel therapeutic possibility to reduce inflammation.

We observed elevated proinflammatory and endothelial dysfunction indices such as BKB1R, ICAM-1, and VWF expression in endothelium following LPS treatment. Tissue plasminogen activator inhibitor 1 (PAI-1) expression which counteracts tissue plasminogen activator (tPA) activity was also significantly increased in LPS-stimulated endothelium confirming the development of impaired fibrinolytic system and endothelium activation, a phenomenon found in patients with severe sepsis [34]. Of interest, endothelium activation and thrombophilia were coincided with the PRCP expression levels in LPS-pretreated endothelium. Incubation of LPS-pretreated endothelium with PK alone resulted in no S2302 (kallikrein substrate) hydrolysis suggesting that the PK activation on LPS-treated HUVEC was not due to PK autoactivation. Our data suggested that there was a causal relationship between LPS-induced endothelium activation and PRCP-dependent PK over-activation.

It has been suggested that the root of vascular disease is due to the increased breakdown of nitric oxide and uncoupling of nitric oxide synthase, the two factors observed in hypertension due to having blunted endothelial vasorelaxation [35,36]. Nonetheless, endothelial dysfunction may involve integrated multi-factorial agents/factors including ROS, angiotensin II, and aldosterone levels [37,38]. PRCP is involved in inflammation pathophysiology, but the intracellular signalling leading to the up-regulation of PRCP is unknown. Therefore, it is likely that upregulation of PRCP expression during inflammatory state might be to sustain vasodilation and promote repair by enhancing liberation of nitric oxide and prostacyclin (PGI2), the two factors generated by the metabolites of PRCP.

**Figure 4:**
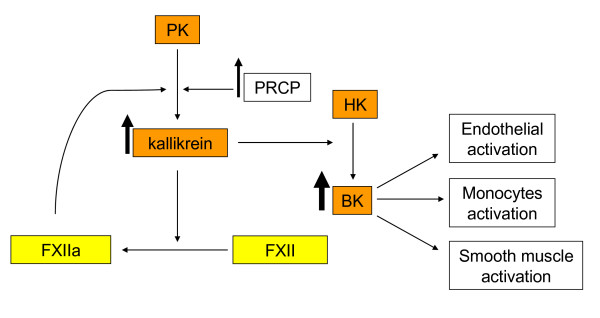
**Schematic representation of the PRCP-dependent PK activation pathway on LPS pretreated endothelial cells**. PRCP: prolylcarboxypeptidase; PK: prekallikrein; HK: high molecular weight kininogen; BK: bradykinin; FXII: factor XII (Hageman factor); FXIIa: activated factor XII. ↑ Indicates an increase, and the thickness of the arrow illustrates the extent of an increase.

During systemic inflammation, uncontrolled activation of PRCP may lead to a robust kallikrein and bradykinin generation which might affect blood vessel integrity. In LPS-treated endothelium, the mRNA for bradykinin B1 receptor was upregulated which may sensitize endothelium to BK mediated permeability. The suppressed activity of angiotensin converting enzyme (ACE) has been documented in cultured endothelial cells during inflammatory challenge [39-41]. ACE inactivates a number of peptide mediators, including BK. If ACE levels are down-regulated, bradykinin levels are higher. The reduced ACE activity feed-forward mechanism forms a vicious circle to amplify and sustain a large flux of BK arising from PRCP-dependent PK activation (Figure 4). PRCP is functionally specialized for hemostasis and its expression might be regulated at the initial phase of acute inflammation.

The bradykinin B 1 receptor (BKB1R) expression was upregulated in LPS-induced endothelial cell activation. BKB1Rs are activated by des-Arg^9^-bradykinin, which is produced from BK by thrombin-activatable fibrinolysis inhibitor (TAFI), carboxypeptidase N and carboxypeptidase M[42,43]. Regardless of the triggers of inflammation, BKB1R is induced only following inflammatory insult. This amplification may be an additional mechanism whereby PRCP promotes a sustained inflammatory response. Of note, PRCP has the ability to metabolize des-Arg^9^-bradykinin in *in vitro *study. Thus, PRCP might influence the balance of BKB2R and BKB1R signaling in endothelial cells by blocking BKB1 receptor-mediated effects. It will be of interest to determine if PRCP plays a pathophysiological role in modulating des-Arg^9^-bradykinin in infection and in other clinically relevant disease situations.

## Conclusion

In conclusion, the data presented in this report supported the idea that PRCP contributed to the initiation of events associated with LPS-stimulated endothelial cells activation. Furthermore, the upregulation of PRCP expression was related to the intensity of kallikrein generation on endothelium (Figure 4). Further investigations must be performed to determine mechanistically how PRCP expression is upregulated in LPS-induced endothelium activation. However, our novel data underline the importance of PRCP in inflammation and in endothelial dysfunction, but its predictive value in inflammation needs to be further investigated.

## Competing interests

The authors declare that they have no competing interests.

## Authors' contributions

NM, MF, KD carried out the experimental work and collected the data. SZ conceived of the study, and participated in its design and coordination and writing the manuscript. All authors read and approved the final manuscript.
